# PUFA-synthase-specific PPTase enhanced the polyunsaturated fatty acid biosynthesis via the polyketide synthase pathway in *Aurantiochytrium*

**DOI:** 10.1186/s13068-020-01793-x

**Published:** 2020-08-31

**Authors:** Sen Wang, Chuanzeng Lan, Zhuojun Wang, Weijian Wan, Qiu Cui, Xiaojin Song

**Affiliations:** 1 CAS Key Laboratory of Biofuels, Shandong Provincial Key Laboratory of Energy Genetics, Shandong Engineering Laboratory of Single Cell Oil, Qingdao Engineering Laboratory of Single Cell Oil, Qingdao Institute of Bioenergy and Bioprocess Technology, Chinese Academy of Sciences, No.189 Songling Road, Laoshan District, Qingdao, 266101 Shandong China; 2 University of Chinese Academy of Sciences, Beijing, 100049 China

**Keywords:** *Aurantiochytrium*, DHA, Thraustochytrids, Polyketide synthase, PPTase

## Abstract

**Background:**

Phosphopantetheinyl transferase (PPTase) can change the acyl-carrier protein (ACP) from an inactive apo-ACP to an active holo-ACP that plays a key role in fatty acids biosynthesis. Currently, the PPTase has been proved to be involved in the biosynthesis of polyunsaturated fatty acids (PUFAs) via a polyketide synthase (PKS) pathway in Thraustochytrids, while its characteristics are not clarified.

**Results:**

Here, the heterologous PPTase gene (*pfaE*) from bacteria was first co-expressed with the PKS system (*orfA*–*orfC*) from Thraustochytrid *Aurantiochytrium*. Then, a new endogenous PPTase (*ppt_a*) in *Aurantiochytrium* was identified by homologous alignment and its function was verified in *E. coli*. Moreover, the endogenous *ppt_a* was then overexpressed in *Aurantiochytrium*, and results showed that the production and proportion of PUFAs, especially docosahexaenoic acid (DHA), in the transformant SD116::PPT_A were increased by 35.5% and 17.6%, respectively. Finally, higher DHA and PUFA proportion (53.9% and 64.5% of TFA, respectively) were obtained in SD116::PPT_A using a cerulenin feeding strategy.

**Conclusions:**

This study has illustrated a PUFAs-synthase-specific PPTase in PKS system and provided a new strategy to improve the PUFA production in Thraustochytrids.

## Background

Polyunsaturated fatty acids (PUFAs), especially docosahexaenoic acid (DHA, 22:6 ω3) and eicosapentaenoic acid (EPA, 20:5 ω3), are rapidly gaining attention, due to their beneficial effects in the cognitive development of infants and their use to reduce the risk of hypertension, cardiovascular diseases, inflammation, and certain cancers [1–4]. Currently, the major commercial source of PUFAs is fish oil; however, several factors such as the reduction of marine fish source, the increasing environmental pollution, and undesirable fishy flavor limit the supply of high-quality PUFAs [5, 6]. Therefore, alternative sources of high-quality PUFAs, especially DHA and EPA, have drawn increasing amounts of attention. Heterotrophic thraustochytrids, are capable of accumulating large of lipids, have been increasing embraced by the market [7, 8].

**Fig. 1:**
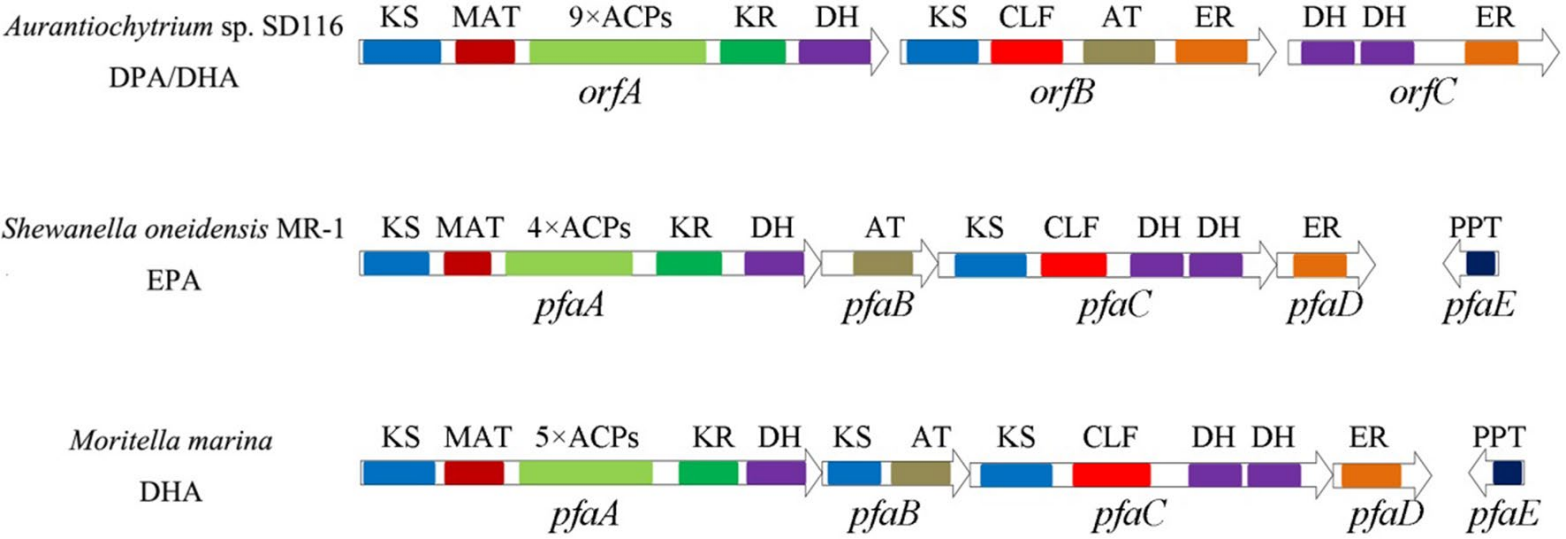
Domain organizations of PKS. KS: β-ketoacyl synthase, MAT: malonyl-CoA transacylase, ACP: acyl-carrier protein, CLF: chain length factor, KR: ketoacyl reductase, AT: acyltransferase, ER: enoyl reductase, DH: dehydratase, PPT: phosphopantetheine transferase

As it is known, PUFAs are synthesized by two pathways, the aerobic desaturase/elongase pathway and the anaerobic polyketide synthase (PKS) pathway [9–11]. Compared with the desaturase/elongase pathway, PKS pathway directly synthesizes the PUFAs from acetyl-CoA and malonyl-CoA substrates with a few intermediates. The PKS pathway is catalyzed by an enzyme complex, and the gene codings for the members of this complex are composed of three or four subunits and possess similar domain structures including β-ketoacyl synthase (KS), chain length factor (CLF), malonyl-CoA transacylase (MAT), acyl-carrier protein (ACP), ketoacyl reductase (KR), acyltransferase (AT), enoyl reductase (ER), and dehydratase (DH) domains (Fig. 1) [8, 12–14]. In addition to the above domains, a phosphopantetheine transferase (PPTase) is also reported to be essential for PUFA synthesis [15]. ACP domain in PKS needs a posttranslational modification to become enzymatically active. PPTase transfers the pantetheine moiety from Coenzyme A to a conserved serine residue of an inactive carrier protein to produce its active form [16, 17]. The gene encoding that PPTase (PfaE) has been identified in several bacterial PKS systems, such as *Shewanella oneidensis* [18] and *Moritella marina* [19], and has been proved to be essential for PUFA synthesis. The putative PPTase encoding gene was also identified in the Thraustochytrid species, such as *Aurantiochytrium limacinum* [20] and *Hondaea fermentalgiana* [7]; however, its characteristics are not clearly clarified.

PPTases can be subdivided into three groups according to their primary sequence and substrate specificity [19, 21, 22]. The name-giving prototype of the first group is AcpS from *Escherichia coli*. The AcpS-type PPTases are 120–140 amino acids in size, act as homotrimers, are associated with primary metabolism in prokaryotes, and accept only ACPs from FAS and ACPs of type II PKS. The second group of PPTases, which can be found in eukaryotes, represents integrated domains of their cognate FAS. PPTases of the third, Sfp-type PPTases, are about twice the size of AcpS, have an extraordinarily broad substrate spectrum, and are active as monomers. Sfp has proven to recognize every carrier protein (CP) tested so far, including PCPs of NRPSs and ACPs of FASs and PKSs [16, 19].

Heterotrophic thraustochytrid, *Aurantiochytrium* sp., has a strong ability to produce biomass and lipid. Particularly, it can produce large amounts of DHA, which comprises nearly 50% of the total fatty acid (TFA) proportion [23–25]. Therefore, *Aurantiochytrium* has become one of the major alternative sources for commercial DHA production.

It is well known that there are two fatty acid pathways in *Aurantiochytrium* [4, 26]. The conventional fatty acid synthase (FAS) pathway is mainly responsible for the synthesis of saturated fatty acids (SFAs), while PUFAs including DHA and docosapentaenoic acid (DPA) in *Aurantiochytrium* are synthesized via the PKS pathway [4, 27]. This PKS system has been confirmed to contain three genes, called *orfA*, *orfB*, and *orfC* (Fig. 1). However, no PPTase gene or PPTase function domain was contained in this PKS system [13, 28].

In the present work, we first confirmed that PPTase was involved in and essential for the PKS system in *Aurantiochytrium* and that the efficiency of DHA synthesis was influenced by overexpression of PPTase. Then, a new endogenous PPTase was identified and overexpressed in *Aurantiochytrium* sp. SD116 to strengthen the PKS system resulting in the improvement of DHA production. The proportion of PUFAs, especially the DHA proportion, had significantly increased in the recombinant SD116::PPT_A strain. The PUFAs proportion was further enhanced by depressing the FAS pathway. Based on this study, a PUFAs-synthase-specific PPTase in PKS system was identified and a new strategy for high PUFA production in *Aurantiochytrium* was provided.

## Results and discussion

### PPTase is essential for the PKS system

**Fig. 2:**
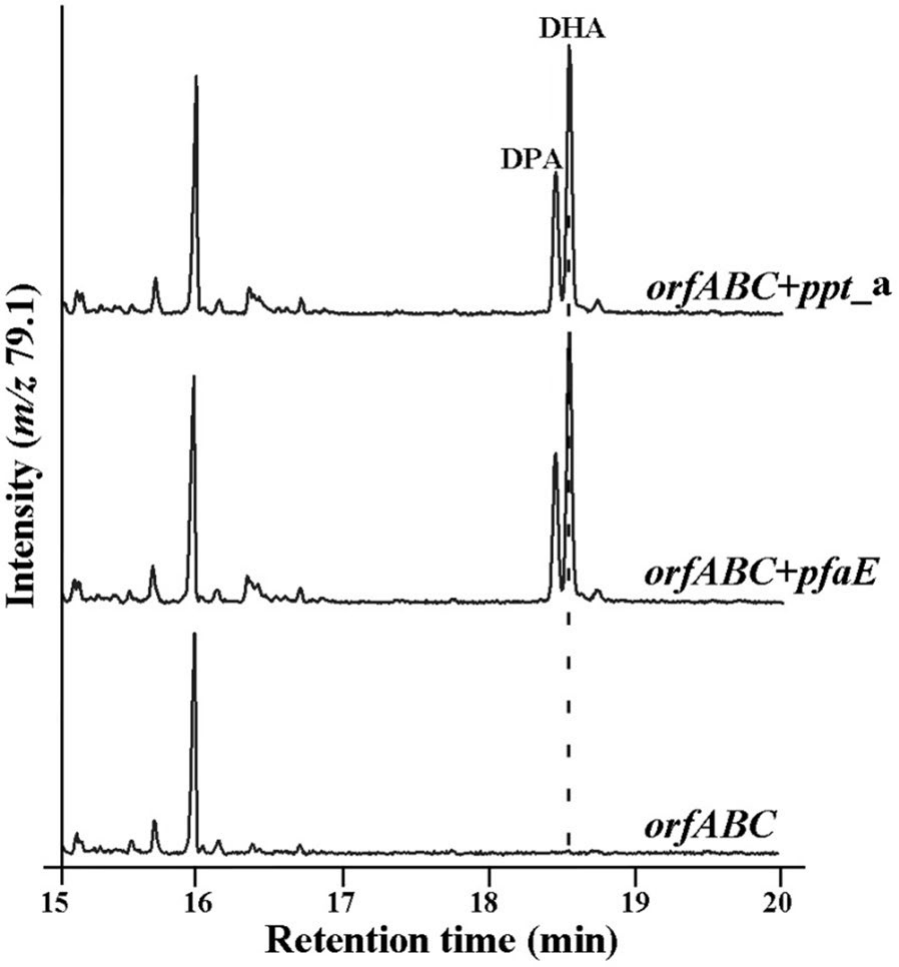
GC–MS analysis traced at m/z 79.1 of products produced in the recombinant *E. coli*. orfABC: *orfA*, *orfB*, and *orfC* were co-expressed in *E. coli*. orfABC + pfaE: *orfA*, *orfB*, *orfC* and *pfaE* were co-expressed in *E. coli*. orfABC + ppt_a: *orfA*, *orfB*, *orfC,* and *ppt_a* from *Aurantiochytrium* were co-expressed in *E. coli*

In thraustochytrid *Aurantiochytrium*, the so-called PKS, can anaerobically synthesize PUFAs de novo. The PKS organization consists of three subunits OrfA, OrfB, and OrfC (Fig. 1). To verify all components of PKS in *Aurantiochytrium*, *orfA*, *orfB*, and *orfC* were cloned and successfully co-expressed in *E. coli*. GC–MS was used to analyze the lipid profiles of *E. coli*, and the result showed that no DHA was produced by the recombinant strain *orfABC* (Fig. 2), suggesting that the heterologous expression of PKS system is not intact. The PKS organization in *Aurantiochytrium* possesses AT, multiple ACPs, MAT, KS, KR, DH, ER, and CLF domains, while no PPTase encoding gene or PPTase domain was identified. The previous studies showed that PPTase is essential for PUFA production in several marine bacteria, and the recombinant product of EPA and DHA in *E. coli* was achieved using the intact PKS gene cluster which contains the PPTase encoding gene from *Shewanella* and *Moritella marina*, respectively [29, 30]. Therefore, we deduced that the PPTase may be also essential for the PKS system of *Aurantiochytrium*. *pfaE* gene, which encodes a PPTase in *Shewanella*, was cloned and co-expressed with *orfA*, *orfB*, and *orfC* in *E. coli*, and the result showed that the recombinant DHA and DPA were produced (Fig. 2). Based on this result, we confirmed that PPTase is involved in and essential for the PKS system in *Aurantiochytrium*.

As functional expression of the PKS requires posttranslational activation by a PPTase, overexpressing PPTase may be beneficial for PUFA synthesis in *Aurantiochytrium*. Then, the *pfaE* gene from *Shewanella* was overexpressed in *Aurantiochytrium* to attempt to improve the DHA production. As shown in Additional file 1, the plasmid pWE-PfaE containing the *pfaE* expression cassette driven by a tubulin promoter (Ptub) and actin transcription terminator (Tactin) was transformed into *Aurantiochytrium* sp. SD116, and the correct transformants named SD116::PfaE were screened by the selection stress of zeocin and further confirmed by genomic PCR. A 0.9-Kbp DNA band was detected in the transformant, but not in SD116 (Additional file 2), suggesting that p*faE* was successfully inserted in SD116.

**Fig. 3:**
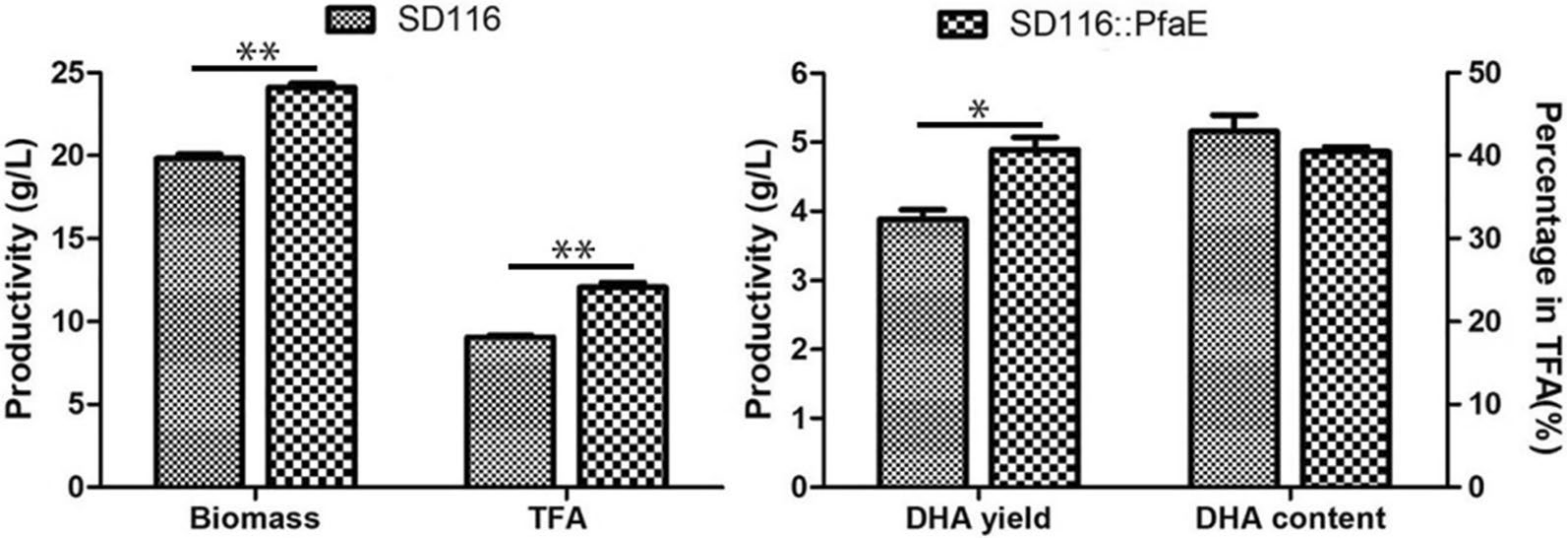
Fermentation profiles in strains SD116 and SD116::PfaE. Biomass and the total fatty acid production in SD116 and SD116::PfaE (left). DHA production and DHA proportion in SD116 and SD116::PfaE (right). **p < 0.01. *p < 0.05

To demonstrate the effects of *pfaE* overexpression, the biomass, lipid accumulation, and fatty acid composition were analyzed. As shown in Fig. 3, the final biomass (dry cell weight) of the transformant SD116::PfaE was 21.6% (*p* < 0.001) higher than that of SD116 at the stationary phase (96 h). In addition, the total fatty acid (TFA) production and the DHA production of the transformant SD116::PfaE were 33.4% (*p* < 0.001) and 25.9% (*p* < 0.05) higher than those in SD116, respectively. However, the DHA proportion (percentage in TFAs) in SD116::PfaE is almost the same to that in SD116 (Fig. 3). Thus, it is evident that overexpression of the PPTase encoding by *pfaE* in *Aurantiochytrium* can significantly improve its ability of fatty acid synthesis (Additional file 3). From these results, we concluded that the PfaE, an Sfp-type enzyme from *Shewanella*, had a relatively broad substrate spectrum which was consistent with the results inferred from its primary structure [17], and it could activate the acyl-carrier protein both from the FAS and PKS pathway in *Aurantiochytrium*.

### An endogenous PPTase was identified in *Aurantiochytrium*

The previous study found that substitution of the PPTase gene from the heterologous species did not influence the function of the PKS pathway producing DHA, but led to different DHA yields [17], suggesting that PPTases are also required specifically to recognize the structure of substrates. There are four repeated ACP domains (for *S. oneidensis*.) or nine (*Aurantiochytrium* sp. SD116) are integrated in the large multifunctional PfaA (or OrfA) product as substrates of PPTases (Fig. 1). Therefore, although different PPTases could lead to PUFA synthesis, they may have a very strict specificity for efficiently activating their substrates. A PPTase which can specifically recognize the nine repeated ACP domains may be better for PUFAs synthesis in *Aurantiochytrium*.

The recombinant DHA was produced in *E. coli* after the *pfaE* was co-expressed with the PKS system of *Aurantiochytrium*, so we deduced that there might be a PPTase in *Aurantiochytrium* involved in the synthesis of PUFAs. A blast search using *S. oneidensis* MR-1 PfaE as probe from the *Aurantiochytrium* sp. SD116 genome yielded one contig encoding a putative PPTase. The putative PPTase (named as PPT_A) contains 271aa (30.5 kDa) with a pI of 5.22, and it exhibits a relatively low similarity (only 24.19%) to the PfaE. To confirm that the PPT_A has PPTase activity, *ppt_a* was cloned and co-expressed with *orfA*, *orfB*, and *orfC* in *E. coli*. As shown in Fig. 2, the recombinant *E. coli* could synthesize DHA. Thus, it is confirmed that PPT_A is a PPTase which can activate the biosynthesis process of PUFAs.

Alignment of representative PfaE [PfaE from *S. oneidensis* (PfaE_S) and *M. marina* (PfaE_M)], PPTase (PPT_M from *A. limacinum* ATCC MYA-1381, PPT_H from *H. fermentalgiana.*), and PPT_A protein sequences revealed the existence of P1, P2, and P3 domains, and the P1 domain was recognized separately as two subdomains of P1a and P1b (Additional file 4). These conserved amino acid domains were defined previous [19, 21]. P2 and P3 are domains participating in Mg^2+^ binding, and P1 (both P1a and P1b) and P3 are involved in substrate (coenzyme A) binding and catalysis [31, 32]. PfaE for EPA and DHA had another conserved sequence of L/VRxL/VLS (P0) [19]. The P0 domain and P1a and P1b domains were predicted to be associated with recognition of the specific tertiary structure of the substrates carrying repeated ACP domains of the PfaA product. However, no similar P0 sequence was identified in PPT_A. Peng et al. alignment of four representative PfaE protein sequences revealed that the P0 domain is not conserved among these four PfaE sequences [17]. The substrate of PPT_A contains nine repeated ACP domains, while the PfaE from *S. oneidensis* and *M. marina* are required to recognize four and five repeated ACP domains, respectively. Therefore, we deduced that the P0 sequences might be conserved for the structure of substrates.

### PPT_A is a PUFA-synthase-specific PPTase in the PKS system of *Aurantiochytrium*

**Fig. 4:**
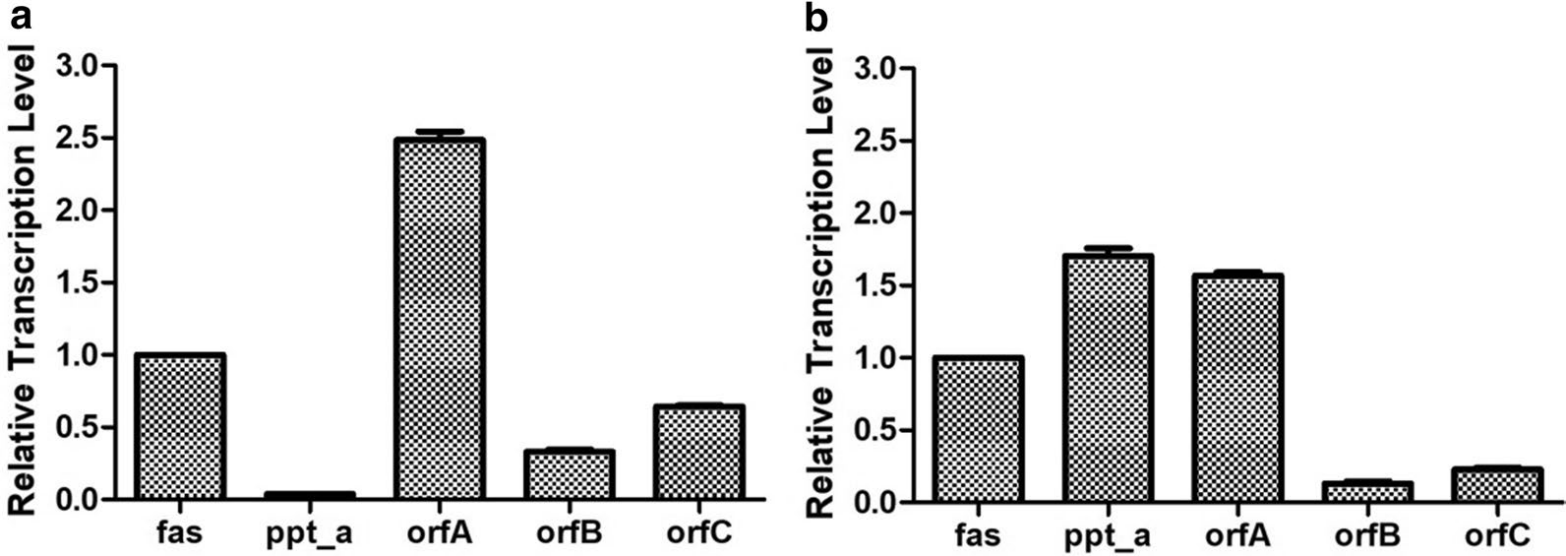
The relative transcription levels of the fatty acid synthesis genes in strain SD116 (**a**) and SD116::PPT_A (**b**). *fas* gene was used as the contrast in each strains

The stated result demonstrated that PPT_A is involved in the PUFA biosynthesis (Fig. 2). However, the transcription level of *ppt_a* in *Aurantiochytrium* sp. SD116 is only 4% of that of *fas* gene at the logarithmic phase of growth, which is obviously lower than other fatty acid synthesis genes (Fig. 4a). Therefore, we deduced that increasing the PPTase activity may improve the lipid and DHA production in *Aurantiochytrium*. Therefore, the *ppt_a* gene was overexpressed in vivo. The expression cassette containing *ppt_a* and *zeo*^R^ resistance gene was transformed into *Aurantiochytrium* via electroporation to obtain SD116::PPT_A strain (Additional file 5). The correct transformants were screened by the selection stress of zeocin and further confirmed by genomic PCR. A 0.9-K bp DNA band was detected in the PCR with the SD116::PPT_A, but not with the wild-type SD116 (Additional file 6), suggesting that *ppt_a* was successfully inserted in SD116.

**Fig. 5:**
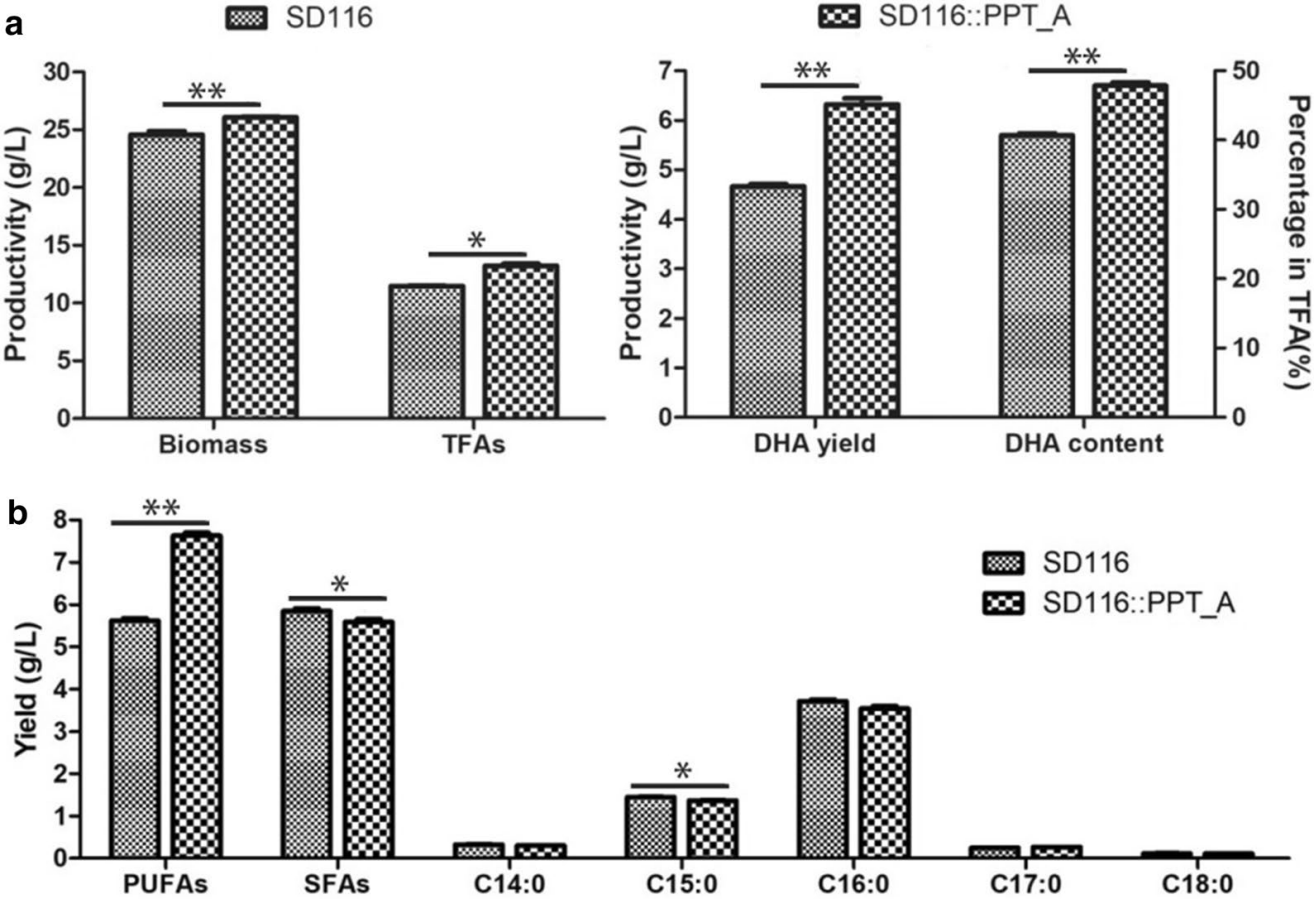
Fermentation profiles in strains SD116 and SD116::PPT_A. **a** Biomass and the total fatty acid production in SD116 and SD116::PPT_A (left). DHA production and DHA proportion in SD116 and SD116::PPT_A (right). **b** Saturated fatty acid (SFA) yields in strains SD116 and SD116::PPT_A. **p < 0.01. *p < 0.05

To further characterize SD116::PPT_A transformant, both the cell biomass and fatty acid profiles were detected. As shown in Fig. 5, the final biomass and TFA production of SD116::PPT_A were 6.1% (*p* < 0.01) and 15.2% (*p* < 0.05) higher than that of SD116, respectively. These results suggest that overexpression of PPT_A can improve the cell growth and lipid synthesis in *Aurantiochytrium*. In addition to the increased biomass and TFA production, the DHA production and DHA proportion of SD116::PPT_A were 35.5% (*p* < 0.001) and 17.6% (*p* < 0.001) higher than that of SD116, respectively (Fig. 5a). Interestingly, although the biomass and TFA production were increased, the total saturated fatty acid (SFA) production of SD116::PPT_A is 95.5% (*p* < 0.05) of that of SD116 (Fig. 5b), suggesting that overexpression of PPT_A has little effect on the SFAs pathway. From these results, we concluded that the newly discovered endogenous PPT_A can specifically activate the PUFA biosynthesis pathway. Due to the successful overexpression of the *ppt_a* gene, the transcription level of *ppt_a* in SD116::PPT_A was 1.70-fold of that of *fas* gene, while the transcription level ratio (*l*_*ppt_a*_ vs *l*_*fas*_, the transcription level of *fas* gene was used as the control, because SFAs’ production had little effect by overexpressing the *ppt_a* in *Aurantiochytrium*) was 42.5 times that of wild type (Fig. 4b). Moreover, the relative transcription ratios of *orfA*, *orfB*, and *orfC* in SD116::PPT_A were significantly decreased. At the same time, the expression levels of these genes in transformant containing empty plasmid did not change significantly compared to wild-type strain (Additional file 7). Therefore, we deduced that the newly discovered endogenous PPT_A played a key role in promoting the efficiency of PUFAs biosynthesis.

### Enhancement of DHA proportion using the cerulenin feeding strategy

**Fig. 6:**
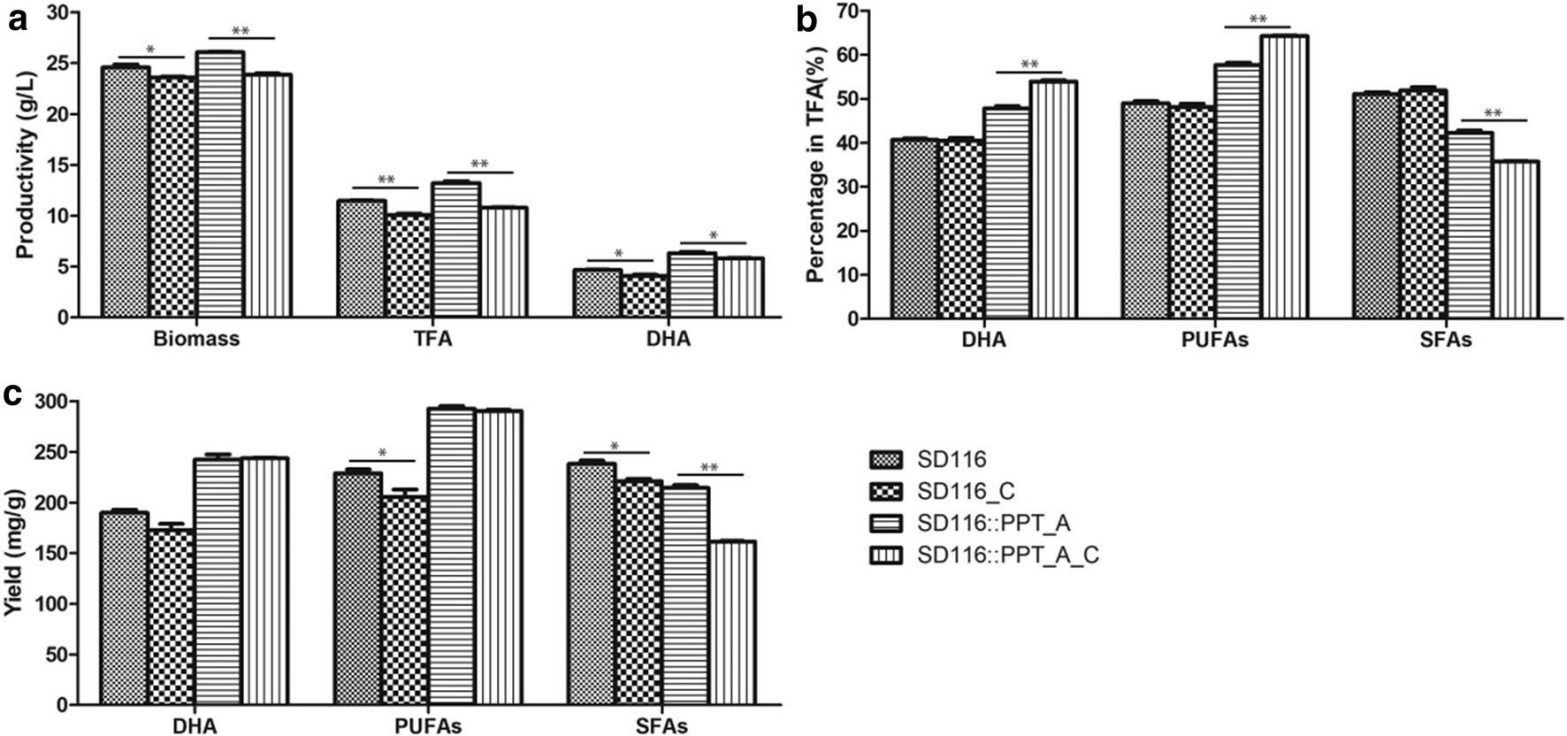
Fermentation profiles of strains SD116 and the mutant SD116::PPT_A upon adding 0.1 mg/L cerulenin (named as SD116_C and SD116::PPT_A_C, respectively). **a** The final biomass, TFA production, and DHA production in SD116, SD116_C, SD116::PPT_A, and SD116::PPT_A_C. **b** The DHA, PUFAs, and SFAs’ proportion (percentage in TFA) in SD116, SD116_C, SD116::PPT_A, and SD116::PPT_A_C. **c** The percentage of DHA, PUFAs, and SFAs (mg/g dcw) in SD116, SD116_C, SD116::PPT_A, and SD116::PPT_A_C. **p < 0.01. *p < 0.05

The endogenous PPT_A can specifically activate the PUFA biosynthesis pathway and has little effect on SFA synthesis pathway. Thus, inhibition of the SFA biosynthesis pathway might further improve the DHA proportion and production. Cerulenin is a fungal antibiotic that irreversibly inhibits the activity of KS domain [33]. Previous study showed that lower concentrations of cerulenin inhibited SFA synthesis pathway, and higher concentrations blocked the PUFA synthesis pathway in *Schizochytrium* [34]. Therefore, 0.1 mg/L cerulenin was used to enhance PKS pathway in *Aurantiochytrium*. As shown in Fig. 6a, the final biomass and TFA production were decreased by 8.4% (*p* < 0.001) and 18.4% (*p* < 0.001) in SD116::PPT_A, respectively, after addition of cerulenin in medium. The DHA production was decreased by 8.0% (*p* < 0.05) when SD116::PPT_A was grown with cerulenin; however, the DHA and PUFAs proportion were increased by 12.7% (*p* < 0.001) and 11.3% (*p* < 0.001), respectively (Fig. 6b). Although cerulenin inhibits the final biomass and TFA production, SD116::PPT_A could produce higher DHA proportion (53.9%) which is beneficial to a high-quality DHA product. As shown in Fig. 6c, the yields of DHA and PUFAs in SD116::PPT_A were 242.5 ± 7.0 mg/g dcw (dry cell weight) and 292.5 ± 3.8 mg/g dcw, respectively. Moreover, these percentages were not significantly changed after addition of cerulenin (243.7 ± 0.9 mg/g dcw and 290.3 ± 1.8 mg/g dcw, respectively), suggesting that cerulenin has little effect on PKS system. The percentage of SFA in SD116::PPT_A decreased by 24.7% (*p* < 0.001) after addition of cerulenin in medium, suggesting that cerulenin inhibited the SFA synthesis pathway more efficiently. Based on these results, we confirmed that low concentrations of cerulenin could selectively inhibit the SFA synthesis pathway in *Aurantiochytrium* SD116::PPT_A.

Both PKS and FAS systems use the same substrate to synthesize fatty acids. Here, we found that inhibiting the FAS pathway could improve the DHA proportion in *Aurantiochytrium*. A high-quality DHA product from SD116::PPT_A was obtained with the cerulenin strategy. However, addition of cerulenin is not conductive to the industrial production of DHA. Further study to down-regulate gene expression of *fas* by genetic engineering technology will provide more advantages for DHA industrial production.

## Conclusions

In this study, a new endogenous PPTase (PPT_A) was identified and proved to be essential to synthesize PUFAs in thraustochytrids *Aurantiochytrium*. Furthermore, the DHA and PUFA proportion in PPT_A overexpressed strain can reach to 53.9% and 64.5% in TFA, respectively. This study has first identified a PUFAs-synthase-specific PPTase in PKS system of *Aurantiochytrium* and provided a new strategy to improve the PUFA production in Thraustochytrids.

## Methods

### Strains and cultivation

*Aurantiochytrium* sp. SD116 was cultivated in a seed liquid medium containing 30 g/L glucose, 10 g/L yeast extract, and 10 g/L artificial sea salt. The strain was cultivated at 25 °C and shaken at 200 rpm for 48 h, and then, 2% (v/v) of cultures were transferred into fermentation medium containing 60 g/L glucose, 20 g/L yeast extract, and 15 g/L sea salt. The screening medium, containing 30 g/L glucose, 10 g/L yeast extract, 5 g/L artificial sea salt, 1.5% agar, and 50 ug/mL zeocin, was used for screening of transformants [4, 35].

### Molecular cloning and plasmid construction

The zeocin expression cassette was amplified from the pGZC-1 [4] with the primers 0426-3F and 0426-3R. The tubulin promoter, the actin terminator, and homologous fragments 18S1 and 18S2 were amplified from *Aurantiochytrium* genome with primer pair 194F/194R, 196F/196R, 191F/191R and 192F/192R, respectively. *pfaE* gene was amplified from *S. japonica* strain KCTC 22435 genome with primers 195F and 195R. The *pfaE* expression cassette was assembled by the overlap PCR, and then ligated to the zeocin expression cassette and 18S1 and 18S2 by overlap PCR (Additional file 1). To construct the *ppt_a* expression cassette, the *ppt_a* gene was amplified from *Aurantiochytrium* genome with primers 197F and 197R which contains the 2A sequence. The plasmid vector was amplified from pGZC-1 with primers 198F and 198R, and then, *ppt_a* gene was ligated to plasmid vector by seamless clone method (Additional file 4). The primer pair 191F/192R was used to amplify the line fragments for expression of *pfaE* and *ppt_a*, and then electrotransformation of *Aurantiochytrium* sp. SD116 was performed according to reported protocol [4].

pACYC Duet-1 and pET Duet-1 were used to express *orfA*, *orfB*, *orfC*, *pfaE*, and *ppt_a*. *orfA*, *orfB*, *orfC*, and *ppt_a* were amplified from *Aurantiochytrium* genome with primer pairs 1911-1F/1911-1R, 1911-2F/1911-2R, 1911-3F/1911-3R, and 1911-4F/1911-4R, respectively. *pfaE* was amplified from *S. japonica* genome with primers 1911-5F and 1911-5R. The plasmid pET Duet-1 was digested with BamH I and Not I, and then ligated with *orfC* by seamless clone method to get pET-D-orfC. The plasmid pET-D-orfC was digested with Nde I and ligated with *orfB* to generate the plasmid pET-D-orfC–orfB. The plasmid pACYC Duet-1 was digested with Nde I and Kpn I and then ligated with *pfaE* by seamless clone method to generate pACYC-D-pfaE. The plasmids pACYC-D-pfaE and pACYC Duet-1 were digested with BamH I and Not I and then ligated with *orfA* to generate the plasmid pACYC-D-pfaE-orfA and pACYC-D-orfA, respectively. The plasmid pACYC-D-orfA was digested with Nde I and ligated with *ppt_a* by seamless clone method to generate the plasmid pACYC-D-orfA-ppt_a. pET-D-orfC–orfB was co-transformed in *E. coli* BL21 with plasmids pACYC-D-orfA, pACYC-D-pfaE-orfA, and pACYC-D-orfA-ppt_a, respectively, to generate the recombinant strains.

### Quantitative real-time PCR (qRT-PCR) analysis

The total RNA was isolated using the Trizol reagent (Thermo Scientific) and miRNeasy Mini Kit (QIAGEN, Germany), and subsequent synthesis of cDNA was achieved using a Revert Aid First Strand cDNA Synthesis Kit (Thermo Scientific). The cDNA was used as the template for qRT-PCR analysis with the primers listed in Additional file 8. PCR was performed using a FastStart Universal SYBR Green Master (ROX). 18S rRNA was used as an internal control to normalize the expression levels. The relative transcription level was calculated using the 2^−ΔΔCt^ method.

### Growth and biomass determination

The biomass was expressed as DCW. 5 mL samples were collected and centrifuged at 9000*g* at 4 °C for 5 min, and then, it was determined by freeze-drying to constant weight at − 50 °C.

### Lipid extraction and fatty acid composition analysis

The total lipid was extracted using a combination of chloroform and methanol (2:1, v/v), and weighed according to our previous report [4]. Then, the lipid was dissolved in chloroform and transformed to the fatty acid methyl esters (FAMEs) by using the method of Cui et al. [4].

FAMEs were analyzed by an Agilent 7890B gas chromatograph coupled with 5975C mass spectrometry. Separation was achieved on an HP-INNOWAX (30 m × 0.25 mm i.d., 0.25 μm) capillary column with helium as the carrier gas at a constant flow rate of 1.0 mL/min. The GC temperature programming was set at 100 °C for 1 min, then increased to 250 °C with increments of 10 °C/min, and held for 10 min. The temperature of injection, transfer line, and ion source were 250, 280, and 250 °C, respectively. The mass scan range was 50–800 m/z and selected ion mode (m/z 79.1) for quantitative analysis. FAMEs were identified by comparing mass spectra of the products with those of authentic ones and by utilizing National Institute of Standards and Technology (NIST) mass spectral library.

### Calculation and statistical analysis

All data are the means of three replicates and reported as the mean ± SE. The significance of differences (*p* < 0.05, *p* < 0.01) was assessed using a *t* test.

## Supplementary information

**Additional file 1: Fig. S1.** Scheme for expression of *pfaE* gene into *Aurantiochytrium* sp. SD116.

**Additional file 2: Fig. S2.** Genomic PCR detection.

**Additional file 3: Table S1.** Fatty acid profiles in strains SD116 and SD116::PfaE.

**Additional file 4: Fig. S3.** Alignment of PfaE with the putative PPTases from *Aurantiochytrium* sp. SD116 (PPT_A). Amino acid residues corresponding to core sequences of P1a, P1b, P2, and P3 domains are underlined. PfaE_S from *S. oneidensis*, PfaE_M from *M. marina*, PPT_M from *A. limacinum* ATCC MYA-1381, PPT_H from *H. fermentalgiana.*

**Additional file 5: Fig. S4.** Scheme for expression of *ppt_a* gene into *Aurantiochytrium* sp. SD116.

**Additional file 6: Fig. S5.** Genomic PCR detection.

**Additional file 7: Fig. S6.** The relative transcription levels of the fatty acid synthesis genes in transformant containing empty plasmid.

**Additional file 8: Table S2.** Primers used in this work.

## Data Availability

All data generated or analyzed during this study are included in this published article and its Additional files 1, 2, 3, 4, 5, 6, 7, 8.

## Abbreviations

PPTase: phosphopantetheinyl transferase
ACP: acyl-carrier protein
PUFAs: polyunsaturated fatty acids
PKS: polyketide synthase
DHA: docosahexaenoic acid
EPA: eicosapentaenoic acid
KS: β-ketoacyl synthase
CLF: chain length factor
MAT: malonyl-CoA transacylase
KR: ketoacyl reductase
AT: acyltransferase
ER: enoyl reductase
DH: dehydratase
TFA: total fatty acid
FAS: fatty acid synthase
SFAs: saturated fatty acids
DPA: docosapentaenoic acid
qRT-PCR: quantitative real-time PCR
FAMEs: fatty acid methyl esters
DCW: dry cell weight
