# Supplementary figures and images for: PUFA-synthase-specific PPTase enhanced the polyunsaturated fatty acid biosynthesis via the polyketide synthase pathway in Aurantiochytrium
Source: Biotechnol Biofuels. 2020 Aug 31;13:152. doi: 10.1186/s13068-020-01793-x (PMC7457351; doi:10.1186/s13068-020-01793-x)

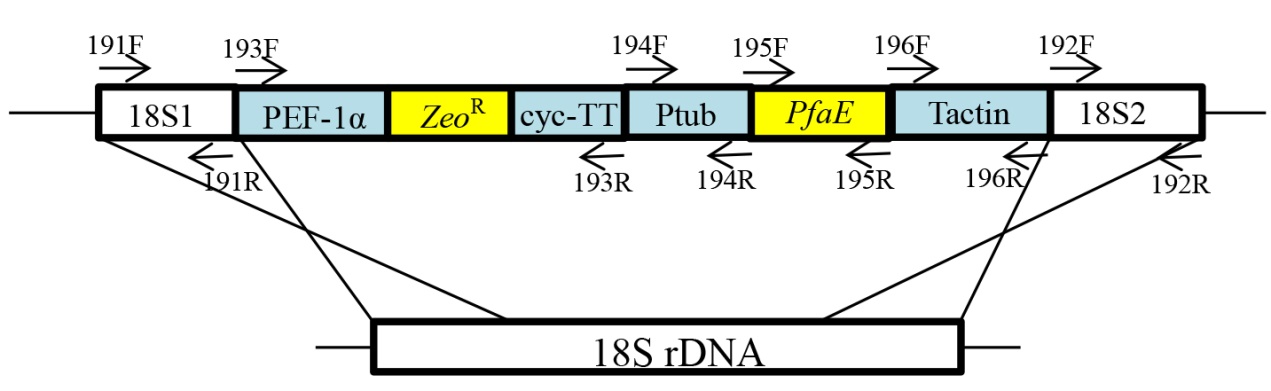


Fig.S1. Scheme for expression of *pfaE* gene into *Aurantiochytrium* sp. SD116.

Supplement: Supplementary file 1 — Additional file 1: Fig. S1. Scheme for expression of pfaE gene into Aurantiochytrium sp. SD116. [file 13068_2020_1793_MOESM1_ESM.docx]

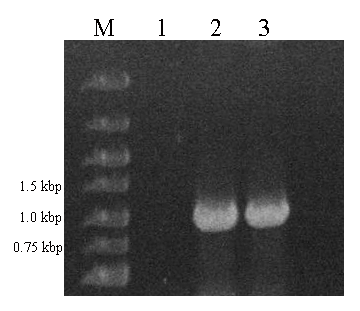


Fig.S2. Genomic PCR detection. Line 1,SD116; line 2, SD116::PfaE; line 3, plasmid control; M, marker.

Supplement: Supplementary file 2 — Additional file 2: Fig. S2. Genomic PCR detection. [file 13068_2020_1793_MOESM2_ESM.docx]

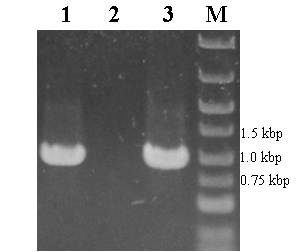


Fig.S5. Genomic PCR detection. Line 1,plasmid control; line 2, SD116; line 3, SD116::PPT_A; M, marker.

Supplement: Supplementary file 6 — Additional file 6: Fig. S5. Genomic PCR detection. [file 13068_2020_1793_MOESM6_ESM.docx]
