# Supplementary material for: PUFA-synthase-specific PPTase enhanced the polyunsaturated fatty acid biosynthesis via the polyketide synthase pathway in Aurantiochytrium
Source: Biotechnol Biofuels. 2020 Aug 31;13:152. doi: 10.1186/s13068-020-01793-x (PMC7457351; doi:10.1186/s13068-020-01793-x)
Supplement: Supplementary file 3 — Additional file 3: Table S1. Fatty acid profiles in strains SD116 and SD116::PfaE. [file 13068_2020_1793_MOESM3_ESM.docx]

Table S1. Fatty acid profiles in strains SD116 and SD116::PfaE.^a^

| Strains | TFA (mg/g DCW) | SFA (mg/g DCW) | PUFA (mg/g DCW) | DHA (mg/g DCW) |
| --- | --- | --- | --- | --- |
| SD116 | 456.7±9.46 | 206.6±16.89 | 250.1±14.53 | 196.1±12.27 |
| SD116::PfaE | 501.0±11.96 * | 250.1±3.60 * | 250.1±10.13 | 202.9±8.51 |

^a^, the weight of fatty acids in dry cell biomass (mg/g DCW). *, P < 0.05. Data are shown as mean ± SD, n=3.
