# Supplementary material for: PUFA-synthase-specific PPTase enhanced the polyunsaturated fatty acid biosynthesis via the polyketide synthase pathway in Aurantiochytrium
Source: Biotechnol Biofuels. 2020 Aug 31;13:152. doi: 10.1186/s13068-020-01793-x (PMC7457351; doi:10.1186/s13068-020-01793-x)
Supplement: Supplementary file 4 — Additional file 4: Fig. S3. Alignment of PfaE with the putative PPTases from Aurantiochytrium sp. SD116 (PPT_A). Amino acid residues corresponding to core sequences of P1a, P1b, P2, and P3 domains are underlined. PfaE_S from S. oneidensis, PfaE_M from M. marina, PPT_M from A. limacinum ATCC MYA-1381, PPT_H from H. fermentalgiana. [file 13068_2020_1793_MOESM4_ESM.docx]

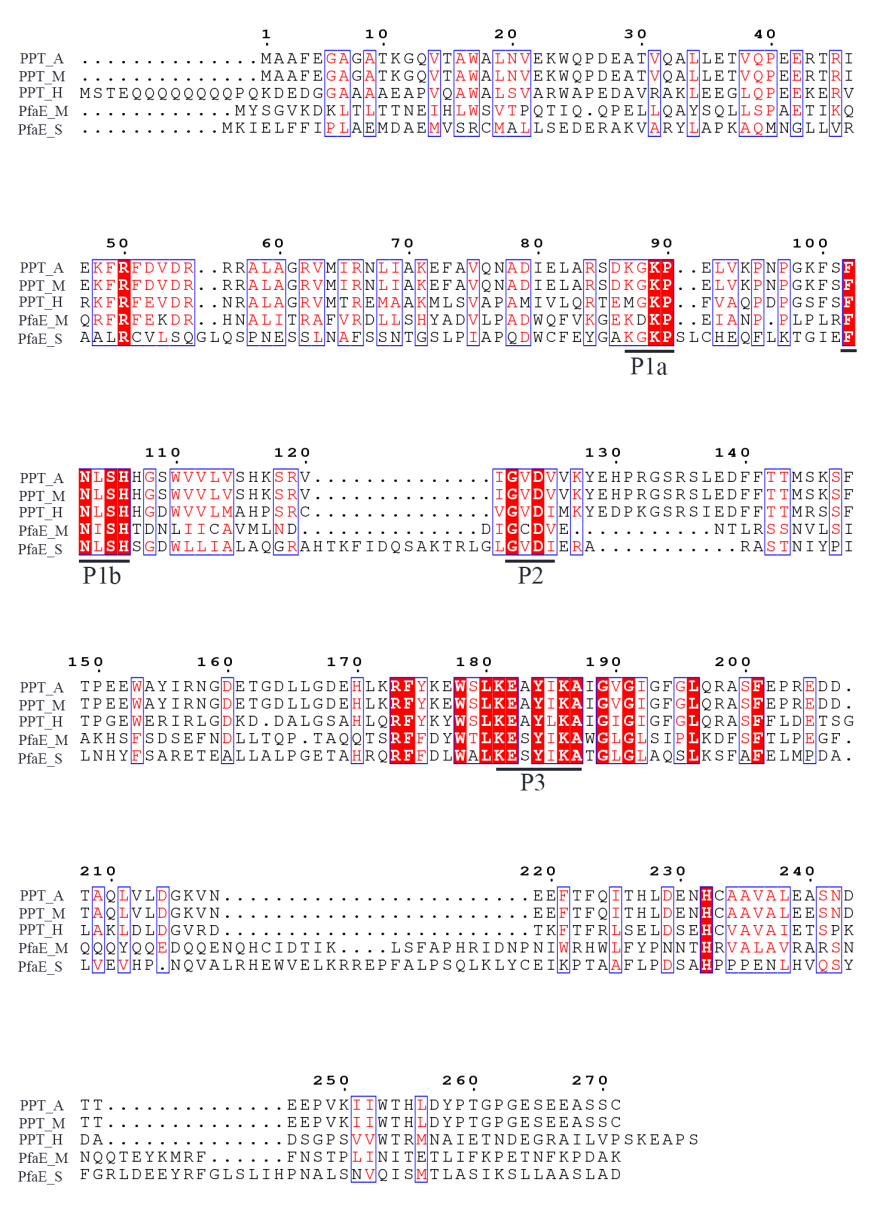


Fig.S3. Alignment of *PfaE* with the putative PPTases from *Aurantiochytrium* sp. SD116 (PPT_A). Amino acid residues corresponding to core sequences of P1a, P1b, P2, and P3 domains are underlined. PfaE_S from *S. oneidensis*, PfaE_M from *M. marina,* PPT_M from *A. limacinum* ATCC MYA-1381, PPT_H from *H. fermentalgiana.*
