# Supplementary material for: PUFA-synthase-specific PPTase enhanced the polyunsaturated fatty acid biosynthesis via the polyketide synthase pathway in Aurantiochytrium
Source: Biotechnol Biofuels. 2020 Aug 31;13:152. doi: 10.1186/s13068-020-01793-x (PMC7457351; doi:10.1186/s13068-020-01793-x)
Supplement: Supplementary file 5 — Additional file 5: Fig. S4. Scheme for expression of ppt_a gene into Aurantiochytrium sp. SD116. [file 13068_2020_1793_MOESM5_ESM.docx]

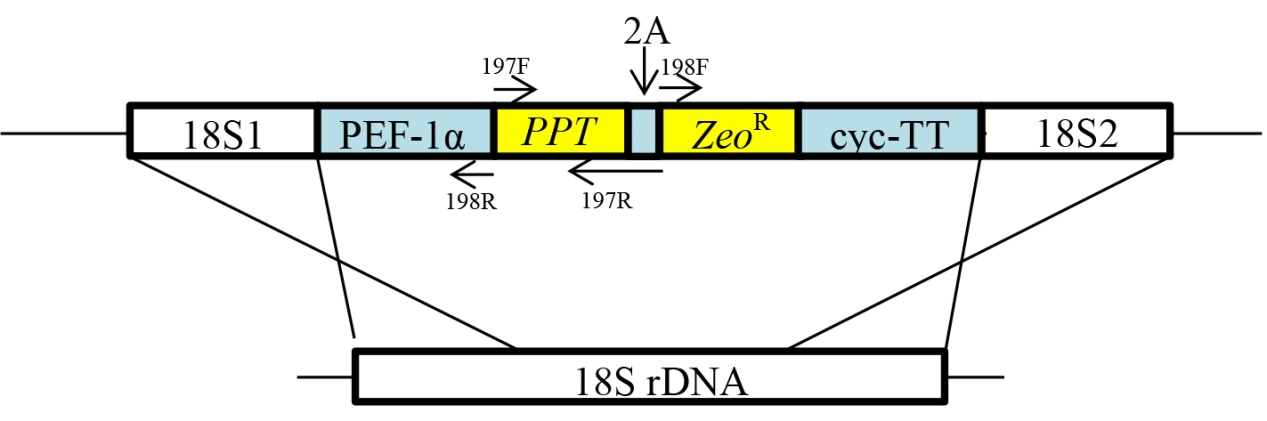


Fig.S4. Scheme for expression of *ppt_a* gene into *Aurantiochytrium* sp. SD116. 2A: the picornavirus’ 2A petide which could self-cleavage at the translation process in eukaryotes.
