# Supplementary material for: PUFA-synthase-specific PPTase enhanced the polyunsaturated fatty acid biosynthesis via the polyketide synthase pathway in Aurantiochytrium
Source: Biotechnol Biofuels. 2020 Aug 31;13:152. doi: 10.1186/s13068-020-01793-x (PMC7457351; doi:10.1186/s13068-020-01793-x)
Supplement: Supplementary file 7 — Additional file 7: Fig. S6. The relative transcription levels of the fatty acid synthesis genes in transformant containing empty plasmid. [file 13068_2020_1793_MOESM7_ESM.docx]

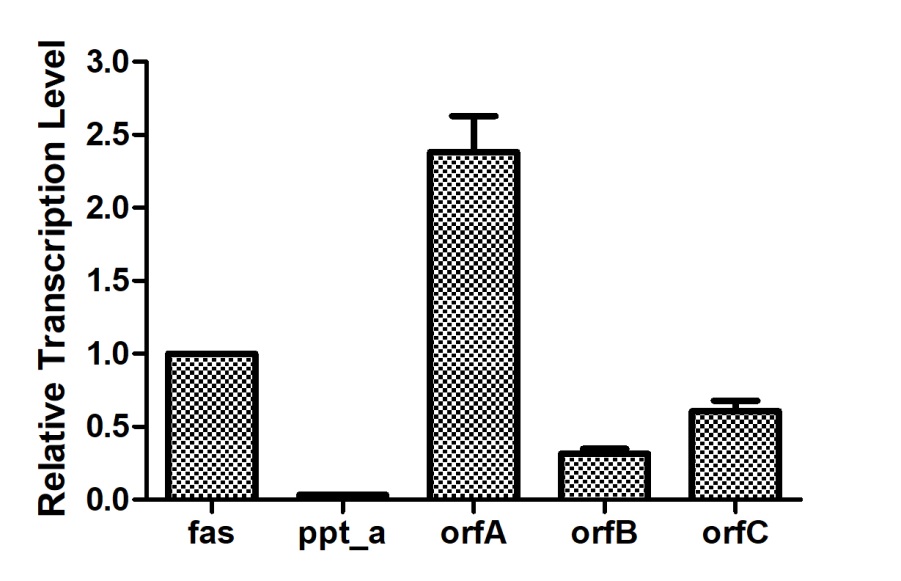


Fig.S6. The relative transcription levels of the fatty acid synthesis genes in transformant containing empty plasmid. *fas* gene was used as the contrast.
