# Supplementary material for: PUFA-synthase-specific PPTase enhanced the polyunsaturated fatty acid biosynthesis via the polyketide synthase pathway in Aurantiochytrium
Source: Biotechnol Biofuels. 2020 Aug 31;13:152. doi: 10.1186/s13068-020-01793-x (PMC7457351; doi:10.1186/s13068-020-01793-x)
Supplement: Supplementary file 8 — Additional file 8: Table S2. Primers used in this work. [file 13068_2020_1793_MOESM8_ESM.docx]

**Table S2. Primers used in this work.**

| Primer | Sequence |
| --- | --- |
| 191F | TAATTCTGGAAATAATACAT |
| 191R | GTGTAGGTTATAAAACTTGATGGTATCTAATCATCTTC |
| 192F | ACGCGGAAGGTCACGCGTAAACGATGCCGACTTGC |
| 192R | TTTCATCGGACCGTTCAATC |
| 193F | AAGTTTTATAACCTACACCCCTGGTGG |
| 193R | TTGCAAATTAAAGCCTTCGAGCGTC |
| 194F | GACGCTCGAAGGCTTTAATTTGCAACAACACTCACAAGCAATCTGC |
| 194R | TTTGCTTGATAGGTTTAAGCTTTAC |
| 195F | GTAAAGCTTAAACCTATCAAGCAAAATGTCTTATTGCTATTATAAATGC |
| 195R | GGGCATTCCATCACTCCAATTCAGTTGGTTTTTATGAACAT |
| 196F | ATTGGAGTGATGGAATGCCCT |
| 196R | GCGTGACCTTCCGCGTGTCAG |
| 197F | AGCTACTAGCACCAAACAAAATGGCGGCCTTCGAAGGTGC |
| 197R | GGTCAACTTGGCCATAGGTCCAGGGTTCTCCTCCACGTCTCCAGCCTGCTTCAGCAGGCTGAAGTTAGTAGCTCCGCTTCCGCAGCTAGATGCCTCTTCAC |
| 198F | GGAAGCGGAGCTACTAACTTCAG |
| 198R | TTTGTTTGGTGCTAGTAGCTTCG |
| 199F | GTGACTTGAACTGTTGCTCCAC |
| 199R | CGGAACGGCACTGGTCAACTT |
| 1911-1F | TCATCACCACAGCCAGGATCCGATGGCTCACCGTGAGAACCG |
| 1911-1R | ACTTAAGCATTATGCGGCCGCTTAGAAGGCAAGGGAGTCAG |
| 1911-2F | AGTTAAGTATAAGAAGGAGATATACATATGGCCTCTCGCAAGAATGT |
| 1911-2R | GATATCCAATTGAGATCTGCCATATGTTACAGGCGCTCAGTGGGCA |
| 1911-3F | TCATCACCACAGCCAGGATCCGATGGCCACTCGCGTGAAGAC |
| 1911-3R | ACTTAAGCATTATGCGGCCGCTTAGAGGGCGTTGGTGGGCT |
| 1911-4F | GGAGATATACATATGATGGCGGCCTTCGAAGGTGC |
| 1911-4R | CTCGAGGGTACCCATATTAGCAGCTAGATGCCTCTTC |
| 1911-5F | GTATAAGAAGGAGATATACATATGATGTCTTATTGCTATTATAAATGCG |
| 1911-5R | TTTACCAGACTCGAGGGTACCTCAGTTGGTTTTTATGAACATTTTTAT |
| PPT-F | GATGTGGACAGGCGAAGAGC |
| PPT-R | TTTGTCGGAGCGTGCTAACT |
| 18S-F | TGCCGACTTGCGATTGTTG |
| 18S-R | TTCAGCCTTGCGACCATACT |
| FAS-F | GCATCTACCACCGTCTTGTTG |
| FAS-R | GGAGCAGAACCAGTCACCTT |
| OrfA-F | AGCCTCCTTGATAGCCTTCTC |
| OrfA-R | TCTGGTGCGTGTTCTTGGT |
| OrfB-F | GTCGGAGTAGGTGGCTTGT |
| OrfB-R | GCCTTCATCGTCACTGGTAC |
| OrfC-F | CTGGTGGTGGTGTTGGATG |
| OrfC-R | GCTGCTTGCGGACATTGT |
